# High-Output Heart Failure in a Patient With Klippel-Trénaunay Syndrome: A Case Report

**DOI:** 10.7759/cureus.38963

**Published:** 2023-05-13

**Authors:** Anna Gubala, Kiran Venkatesh, Mohammed Akhter, Theo E Meyer, Timothy P Fitzgibbons

**Affiliations:** 1 Internal Medicine, University of Massachusetts Medical School, Worcester, USA; 2 Internal Medicine, Sanger Heart and Vascular Institute, Charlotte, USA; 3 Internal Medicine, Duke University School of Medicine, Durham, USA

**Keywords:** pik3ca-related overgrowth spectrum disorder, phosphatidylinositol-3-kinase, klippel-trenaunay, high output, heart failure

## Abstract

Klippel-Trénaunay syndrome (KTS) is a rare and complex congenital syndrome defined as the triad of cutaneous capillary malformation, bone and soft tissue hypertrophy, and venous and lymphatic malformations. KTS is thought to be due to a somatic mutation in phosphatidyl-inositol 3 kinase. It belongs to a group of syndromes termed the PI3CA-Related Overgrowth Spectrum (PROS) disorders. Because of the rarity and clinical heterogeneity of these disorders, management is patient specific, and best evidence guidelines are lacking. The most common clinical complications are thromboembolism, thrombophlebitis, pain, bleeding, and high-output heart failure. Surgery is recommended for hemangiomas and chronic venous insufficiency. The early identification of children with PROS disorders has allowed treatment with mTOR inhibitors which have been shown to be effective. The recent development of a direct PI3K inhibitor (alpelisib) has shown promise in preventing abnormal growth and long-term complications of KTS. This report documents a case of high-output heart failure due to the vascular malformations associated with KTS in a 57-year-old male patient and discusses current literature regarding the management of KTS with inhibitors of mTOR and PI3KCA.

## Introduction

Klippel-Trénaunay Syndrome (KTS) is a rare congenital syndrome associated with a triad of cutaneous capillary malformations, bone and soft tissue hypertrophy, and venous and lymphatic malformations [1]. Incidence is estimated at two to five per 100,000 and is reported more often in males than in females [2]. There is no racial predilection that has been observed [2]. Most often it is a sporadic condition; however, rare familial cases have been reported [3].

Characteristic features of KTS include cutaneous capillary malformations (“port wine stains”) as well as asymmetric hypertrophy of bones and soft tissues. Port wine stains, varicosities, and bone and soft tissue hypertrophy are seen in 98%, 72%, and 94% of cases, respectively [4]. Limb abnormalities manifest as increased length due to bone hypertrophy and increased girth due to soft tissue hypertrophy. Clinically, patients commonly have pain and lymphedema, typically unilateral and affecting the lower extremities [2]. Some psychiatric complications, including depression and anxiety, are also associated with KTS [5]. Other presentations include varicose veins, which are present at birth and may affect extensive areas of the extremities, as well as deep venous system hypoplasia, agenesis, atresia, duplication, and abnormal valve formation. Vascular malformations can also involve visceral organs such as the spleen, liver, pleura, bladder, and colon resulting in internal hemorrhage manifesting as hematuria or hematochezia [2]. KTS patients are at risk for deep venous thromboses and pulmonary embolisms due to aberrancies in venous blood flow, and all pregnant KTS patients are recommended to receive anticoagulation [6]. High-flow arteriovenous fistulas are uncommon in KTS. The presence of such fistulas is more consistent with Parkes-Weber syndrome [7]. However, in a cohort of 47 pediatric KTS patients at the Mayo Clinic 13% presented with high-output heart failure [8]. Herein, we report an adult male who presented to the cardiology service with new onset high-output heart failure.

## Case presentation

A 57-year-old male with a history of KTS presented to our institution with right lower extremity swelling that had progressed to his right flank, buttocks, and abdomen, with associated dyspnea on exertion and pain in his right lower extremity. His past medical history is significant for a right below-the-knee amputation due to chronic lower extremity wounds, right femoral artery aneurysm status post superficial femoral artery to popliteal artery bypass with Teflon graft, and recent angiogram revealing proximal 75% stenosis at the distal anastomosis with a large arterial-venous malformation just distal to the anastomosis.

On exam, the patient was hypertensive 132/93 mmHG and tachypneic (16 breaths per minute) but otherwise hemodynamically stable. Cardiovascular examination revealed a regular rate and rhythm with no murmurs, JVD extending to 5 cm above the sternal notch, and pitting edema bilaterally to the abdomen. Skin exam was notable for a large lateral varicosity in the right lower extremity, with a small erythematous wound at the distal amputation stump site. The remainder of his exam, including the left lower extremity, was unremarkable.

Prior to the time of presentation, the patient had already undergone a significant workup of his KTS and vascular imaging of his lower extremity. Since birth, he had port wine stains and hypertrophy of his right lower extremity. He had undergone multiple prior surgeries, including growth plate removal from his right leg to equalize leg length and right superficial femoral artery to above the knee popliteal artery bypass graft due to aneurysmal degeneration of the femoral artery. He had chronic non-healing ulcers on his RLE.

He then underwent a right below-the-knee amputation. Gross pathology of the limb revealed phlebosclerosis and dilatation with subcutaneous hemangiomas, chronic ulceration, and dermal fibrosis. Major arterial vessels were patent but revealed foci of medial calcification. Organizing venous thrombosis was seen within the ankle.

A repeat angiogram five years later revealed stenosis at the distal end of the SFA to popliteal PTFE graft and post-stenotic dilatation. The graft was resected, and a new longer interposition graft was placed. Several years later, he underwent angioplasty of the distal and proximal anastomoses of the femoral-popliteal bypass graft due to increased velocities at the proximal and distal anastomoses and claudication of the stump.

He underwent a repeat angiogram which revealed significant stenosis of the right femoral to popliteal artery bypass graft, a large AV malformation fed by the deep femoral artery, substantial elongation and dilatation of the entire right iliac and common femoral arteries, and significant stenosis of the graft. No intervention at that time due to the substantial amount of blood flow to the limb. It was at this time the patient presented to our care. There was no family history of KTS or other vascular disorders. The patient had never had genetic testing.

Blood work revealed a BNP of 1,268 pg/mL and creatinine of 1.06 mg/dL. Hemoglobin was 13.3 gm/dL and hematocrit was 41.8%. Platelets were 200,000 per mcL. EKG showed normal sinus rhythm, right axis deviation, poor R-wave progression, right atrial enlargement, nonspecific intraventricular conduction delay, grossly unchanged from prior, and no ischemic changes.

Transthoracic echocardiography demonstrated a dilated left ventricular with low normal systolic function (estimated EF 50%-55%) with possible basal inferior hypokinesis. There was concentric LV hypertrophy. The RV was mildly dilated and hypokinetic. The left atrium was markedly dilated (LA volume index 84 mL/m^2^) and there was mild pulmonary hypertension (estimated PASP 53 mmHg).

**Figure 1:**
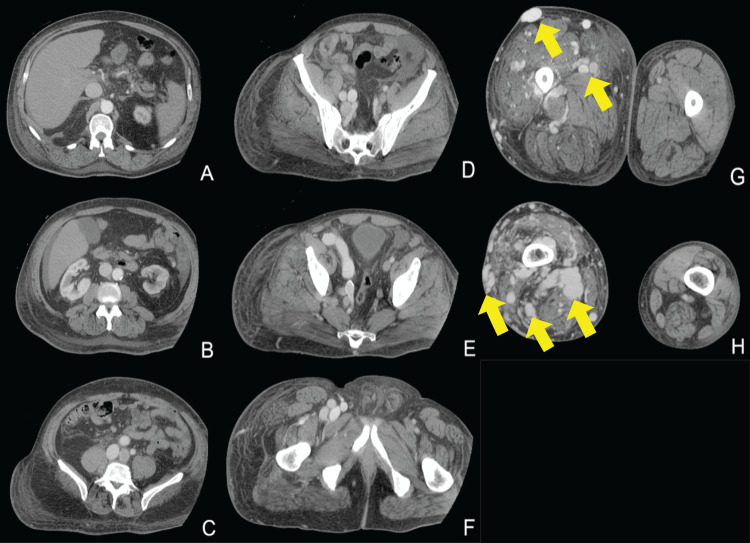
CT angiogram of the right leg. The prominent arterial malformations are best seen in panels G and H (yellow arrows).

**Figure 2:**
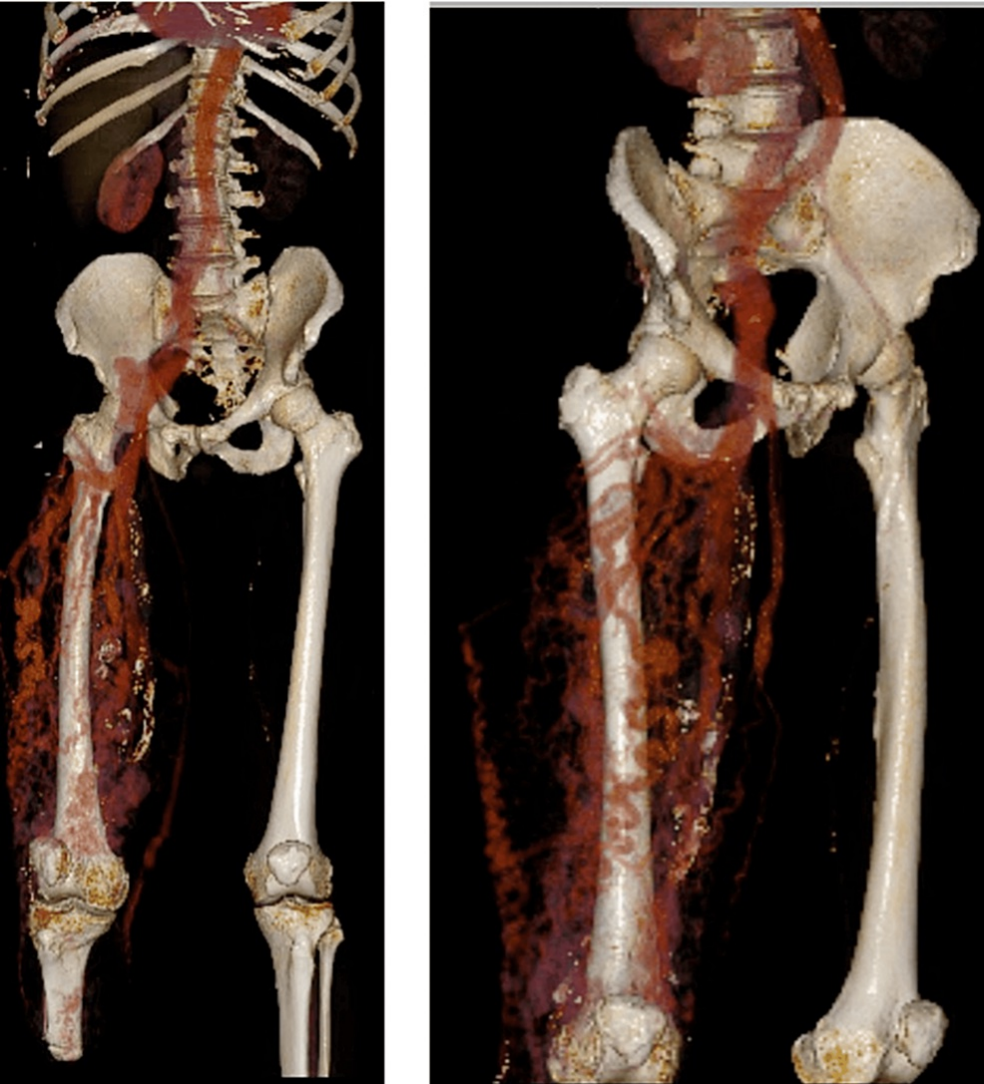
3D reconstruction of the arterial malformations in the right leg. The right femoral-popliteal bypass graft can be seen in the medial aspect of the thigh.

CTA chest showed trace right pleural effusion but otherwise, no acute abnormality. CTA abdomen and pelvis showed diffuse right lower extremity arteriovenous malformation consistent with known KTS, as well as perihepatic ascites and mild right pleural effusion (Figures 1A-1H, 2).

The patient was initially admitted to the vascular medicine service. There was no clear vascular intervention to be done. He was treated with furosemide and lisinopril with an improvement in edema. His leg was very painful, and he was started on acetaminophen and oxycodone. He was discharged home to follow up in the cardiology clinic.

Given the new presentation of heart failure with preserved ejection fraction, the initial differential diagnosis included myocardial ischemia, restrictive or hypertrophic cardiomyopathy, or high-output heart failure to excessive AV shunting. A thallium pharmacologic nuclear stress test was performed to exclude ischemic coronary disease. The test was negative for chest pain, without ischemic ECG changes and negative for any arrhythmias. Wall motion was normal, and the ejection fraction was estimated to be 55%. There were no perfusion defects.

In the setting of the known vascular malformations and TTE findings there was suspicion for high-output heart failure. After multidisciplinary consultation it was decided to do a left heart and right heart catheterization with transient balloon occlusion of the right iliac artery. Left heart catheterization showed no significant coronary artery disease. Right heart catheterization showed evidence of high-output heart failure, with a very high CO and low pulmonary artery and wedge pressures (Table 1).

**Table 1:** Hemodynamic measurements during cardiac catheterization at baseline and after occlusion of right iliac artery RA (Right atrium), PA (Pulmonary Artery), PCWP (Pulmonary Capillary Wedge Pressure), Ao (Aorta), LV (Left ventricle), CO (Cardiac output), CI (Cardiac Index), TD (Thermodilution).

Right heart catheterization		
	Baseline (mmHg)	After occlusion of right iliac
RA	16	--
PA	42/19 (27)	56/21 (36)
PCWP	18 (v waves to 30)	24 (v waves to 40)
Left heart catheterization		
Ao	88/44 (60)	--
LV	88/15	--
Fick CO/CI	8.80 L/min / 3.90 L/min/m2	6.77 L/min / 3.0 L/min/m2
TD CO/CI	9.53 L/min / 4.22 L/min/m2	7.10 L/min / 3.1 L/min/m2
RA SaO2	77%	--
PA SaO2	78%	72%
Ao SaO2	94%	94%

With occlusion of the right iliac artery, the Fick CO decreased from 8.80L/min to 6.77 L/min and the SVR rose to greater than 600 mmHg⋅min⋅mL-1.

The patient was managed as an outpatient for chronic heart failure and severe pain. He developed new onset atrial fibrillation and was started on apixaban. Amputation at the hip was not considered for two reasons. Despite pain and class 2 heart failure symptoms, the patient was quite functional with his right lower extremity prosthesis. Second, the surgery would be very high risk due to extensive AV malformations in his right thigh and would require hip disarticulation. After continued follow up with cardiology and vascular surgery, the patient was referred to interventional radiology for staged embolization of the AV fistulas using a liquid embolic system comprised of ethylene vinyl-alcohol copolymer, DMSO dimethyl-sulfoxide and TA micronized tantalum powder (Onyx). He had two procedures and tolerated them well. Prior to a third procedure, the patient died suddenly at home of unknown causes.

## Discussion

High-output heart failure

The vascular malformations of KTS can lead to lowered systemic vascular resistance (SVR), excessive vasodilation, and high flow states. These high-flow states, if left unchecked, can progress to high-output heart failure. Reddy et al. published a retrospective analysis of patients referred to the Mayo Clinic catheterization laboratory for hemodynamic assessment between 2000 and 2004 and found that 23% of them had high-output heart failure from arterio-venous shunts, which included congenital, traumatic, or hemodialysis fistulas [9].

Compared to controls, patients with high-output heart failure had eccentric left ventricular remodeling, greater natriuretic peptide activation, higher filling pressures, pulmonary hypertension, and increased cardiac output (CO) despite similar ejection fraction. The elevated CO was related to both lower arterial afterload (decreased SVR) and higher metabolic rate (increased oxygen consumption). Mortality was increased compared to controls (HR: 3.4, 95% CI 1.6-7.6). Excessive vasodilation was associated with the poorest prognosis: patients with low SVR (bottom quartile, <1,030 dyne*m2/s*cm5) had increased mortality compared to patients with mildly depressed or normal SVR (61% vs 36%, HR: 2.5, CI: 1.2-1.5, p=0.01) [9]. Increased vasodilation and arteriovenous shunting lead to decreased SVR. This in turn causes an increase in CO, as well as arterial underfilling which leads to renal hypoperfusion and in turn plasma volume expansion. Standard therapies for heart failure including diuretics, vasodilators, and inotropes, are potentially harmful in high-output heart failure, making treatment complicated [9].

Mechanism of KTS

Due to its sporadic incidence, the mechanism of KTS was unknown for a long time. Theoretical mechanisms behind the overgrowth were thought to involve venous atresia and chronic venous hypertension leading to stasis, edema, varicosities, and hypertrophy. It is hypothesized that these changes cause increased blood flow in an already abnormal capillary network, cutaneous venous channels causing overgrowth in fetal life, defective remodeling of vessels during embryogenesis, and mesodermal defects inducing hemangiomas and varicose veins [2]. The persistence of an embryological vascular system may contribute, but this would not fully explain all the possible associated conditions of KTS [10]. A generalized developmental mesodermal anomaly, acting primarily on angiogenesis, would further explain the features of KTS [11].

Recently, KTS has been classified to the PI3KCA-related overgrowth spectrum (PROS) [12]. Phosphatidylinositol 3-kinase (PI3K) is a key lipid kinase that controls signaling involved in cell proliferation, motility, survival, and metabolism. PI3K acts via phosphatidylinositol bis- and triphosphate (PIP2 and PIP3) to activate AKT, activating mammalian target of rapamycin (mTOR). mTOR, which stimulates protein translation, leading to cell growth, vascular proliferation, survival, and angiogenesis [1,13].

The PI3KCA gene encodes a catalytic subunit of the PI3K enzyme. PROS is caused by post zygotic, mosaic, gain of function somatic mutations in the PI3KCA gene, resulting in mosaic PI3KCA activation, which drives the various patient phenotypes [1,14]. The stage of development, the cells originally affected, and the types of mutations determine the severity and phenotypic characteristics of the disease in any given individual. It has been postulated that complete heterozygosity of the mutation would be either lethal to the affected individual or the person would be incapable of genetic transmission of the mutant allele [1].

Prior to KTS classification as part of PROS, management was mainly focused on alleviating symptoms. Due to the variable presentation, there is no consensus for management, apart from that it should be individualized to each patient. Most conservatively, compression stockings, frequent leg elevation, and strict hygiene are used in conjunction with analgesics, antibiotics, and corticosteroids for treatment of cellulitis, thrombophlebitis, and associated pain [2]. Additional supportive care includes sclerotherapy and psychological and nutritional support. Surgery is reserved for symptomatic cases, as symptoms may worsen after multiple ligation or stripping procedures [2]. Surgery may be considered in cases of skin ulcerations leading to persistent or recurrent bleeding, digital deformities leading to functional disabilities, or limb overgrowth leading to functional and psychologic impairment. Persistent hematochezia, hematuria, vaginal or esophageal bleeding are also indications to consider surgery [15]. The clinical heterogeneity of the spectrum of disease leads to challenges in establishing management recommendations, which must be based on patient specific considerations [16].

New therapies: Sirolimus and alpelisib

Recent therapies for KTS have targeted the PI3K pathway. Sirolimus, an mTOR inhibitor, acts within the PI3K-AKT-mTOR signaling pathway as described above. In 2011, six children with treatment-resistant venous and/or lymphatic malformations were treated with sirolimus. They experienced substantial size reduction in vascular lesions and symptom improvement, indicating sirolimus’ potential as salvage therapy in patients with refractory disease [17]. Yuan et al. reported a case of a 43-year-old woman with KTS who was successfully treated with sirolimus for a treatment refractory abdomino-pelvic venous malformation that caused chronic debilitating pain. In addition to improvement in pain scoring, surveillance imaging showed reduction of her venous malformation which were persistent at one year follow up suggesting treatment response to continuous sirolimus therapy [18].

Freixo et al. performed a systematic literature review of the data from 73 articles on PubMed on the effects of sirolimus in management of vascular anomalies. This was not specific to KTS patients, and systematic evaluation of outcomes is limited by the broad clinical manifestations of the diverse disease processes of the different vascular anomalies and the anatomic area affected. It was also limited by broad heterogeneity in the definition of outcomes and the evaluation of clinical, hematologic, and radiologic response. However, it did show that in patients with venous malformations, sirolimus was associated with clinical benefit in most patients, with lesion size decrease in 88.9% of patients. In patients with lymphatic anomalies, oral sirolimus treatment was associated with clinical benefit in 94.9% of patients with decrease of lesion size. The most frequently reported adverse effects were oral mucositis (31.9%), dyslipidemia (16.5%), leukopenia (12.3%), gastrointestinal symptoms (10.2%), rash/eczema (8.2%), and infectious complications (5.5%) [19].

In an investigation of safety and efficacy of low dose sirolimus in PROS, Parker et al. found that sirolimus led to a significant change of total tissue volume at affected sites, but not at unaffected sites. However, sirolimus has a high rate of adverse effects, including infection, blood and lymphatic disorders, neutropenia, interstitial pneumonitis, and sirolimus hypersensitivity syndrome, and risks and benefits should be considered for each individual patient prior to initiation [20]. In summary, inhibition of mTOR with agents like sirolimus seems to improve lymphatic and venous malformations in some patients but is associated with many side effects [14].

Recently, new data have shown that direct inhibition of PI3KCA improved symptoms of KTS with a beneficial side effect profile. In a retrospective study of 57 patients (18 adults and 39 children) with PROS, treatment with alpelisib, an orally available direct inhibitor of PI3KCA used in oncology, induced improvement in pain, fatigue, vascular malformation, limb asymmetry, and coagulopathy in most patients at 24 weeks [12]. The most common adverse effects noted in the study were hyperglycemia, aphthous ulcers, and stomatitis [12]. In another study, nineteen patients with PROS and CLOVES syndrome (congenital lipomatous overgrowth, vascular malformations, epidermal naevi, scoliosis, skeletal and spinal syndrome) were treated with alpelisib (BYL719) and a significant improvement in disease symptoms was seen in all patients [14]. Specifically, intractable vascular tumors became smaller in circumference, congestive heart failure was improved as noted on BNP, CO, and left ventricular mass index to body surface area measurements, and hemihypertrophy was reduced. Twelve of the patients had previously undergone debulking surgery, and eight patients had previously received rapamycin treatment for 18 months without clinical or radiological improvement. Adverse effects of treatment included mouth ulcerations which spontaneously resolved, transient hyperglycemia in one patient, and increased insulin requirements for one diabetic patient [14]. Based on these and other studies, the FDA approved alpelisib for the treatment of adult and pediatric patients over two years old with severe PROS manifestations. The results of an ongoing randomized trials are awaited before its use becomes routine; however, there seems to be great potential in the use of PI3KCA inhibitors such as alpelisib in the management of KTS.

## Conclusions

KTS is a rare congenital disease within the PROS classification of disorders characterized by vascular malformations. These malformations when large enough can cause alterations in blood flow that can lead to high-output heart failure, a condition marked by increased morbidity and mortality. Sirolimus and alpelisib have shown promise as potential treatments for KTS but require further investigation and randomized clinical trial testing to elucidate safety and efficacy. Initiation of these agents at the time of diagnosis may reduce the long-term cardiovascular morbidity and mortality of KTS.
